# Genetic basis for denitrification in *Ensifer meliloti*

**DOI:** 10.1186/1471-2180-14-142

**Published:** 2014-06-02

**Authors:** Maria J Torres, Maria I Rubia, Teodoro Coba de la Peña, José J Pueyo, Eulogio J Bedmar, María J Delgado

**Affiliations:** 1 Estación Experimental del Zaidin, Consejo Superior de Investigaciones Científicas (CSIC), P.O. Box 419, 18080 Granada, Spain; 2 Instituto de Ciencias Agrarias, Consejo Superior de Investigaciones Científicas (CSIC), Serrano 115-bis, 28006 Madrid, Spain

**Keywords:** Cu-containing nitrite reductase, Nitrate respiration, Nitric oxide reductase, Nitrous oxide reductase, Periplasmic nitrate reductase

## Abstract

**Background:**

Denitrification is defined as the dissimilatory reduction of nitrate or nitrite to nitric oxide (NO), nitrous oxide (N_2_O), or dinitrogen gas (N_2_). N_2_O is a powerful atmospheric greenhouse gas and cause of ozone layer depletion. Legume crops might contribute to N_2_O production by providing nitrogen-rich residues for decomposition or by associating with rhizobia that are able to denitrify under free-living and symbiotic conditions. However, there are limited direct empirical data concerning N_2_O production by endosymbiotic bacteria associated with legume crops. Analysis of the *Ensifer meliloti* 1021 genome sequence revealed the presence of the *napEFDABC*, *nirK*, *norECBQD* and *nosRZDFYLX* denitrification genes. It was recently reported that this bacterium is able to grow using nitrate respiration when cells are incubated with an initial O_2_ concentration of 2%; however, these cells were unable to use nitrate respiration when initially incubated anoxically. The involvement of the *nap*, *nirK*, *nor* and *nos* genes in *E. meliloti* denitrification has not been reported.

**Results:**

*E. meliloti nap*, *nirK* and *norC* mutant strains exhibited defects in their ability to grow using nitrate as a respiratory substrate. However, *E meliloti nosZ* was not essential for growth under these conditions. The *E. meliloti napA*, *nirK, norC* and *nosZ* genes encode corresponding nitrate, nitrite, nitric oxide and nitrous oxide reductases, respectively. The NorC component of the *E. meliloti* nitric oxide reductase has been identified as a *c*-type cytochrome that is 16 kDa in size. Herein, we also show that maximal expression of the *E. meliloti napA*, *nirK, norC* and *nosZ* genes occurred when cells were initially incubated anoxically with nitrate.

**Conclusion:**

The *E. meliloti napA*, *nirK, norC* and *nosZ* genes are involved in nitrate respiration and in the expression of denitrification enzymes in this bacterium. Our findings expand the short list of rhizobia for which denitrification gene function has been demonstrated. The inability of *E. meliloti* to grow when cells are initially subjected to anoxic conditions is not attributable to defects in the expression of the *napA*, *nirK, norC* and *nosZ* denitrification genes.

## Background

Denitrification is the respiratory reduction of nitrate or nitrite to the gaseous products nitric oxide (NO), nitrous oxide (N_2_O), or dinitrogen (N_2_). N_2_O is a powerful greenhouse gas (GHG) that has a 300-fold greater global warming potential than CO_2_ based on its radiative capacity and could persist for up to 150 years in the atmosphere [IPCC 2007, [1]]. In bacteria, the denitrification process requires four separate enzymatically catalysed reactions. The first reaction in denitrification is the reduction of nitrate to nitrite, which is catalysed by a membrane-bound nitrate reductase (Nar) or a periplasmic nitrate reductase (Nap) (reviewed in [2-6]). In denitrifying bacteria, the reduction of nitrite to nitric oxide is catalysed by two types of respiratory Nir: the NirS *cd*_1_ nitrite reductase, a homodimeric enzyme with haems *c* and *d*_1_, and NirK, a copper-containing Nir [7-11]. Then, nitric oxide is reduced to nitrous oxide by three types of nitric oxide reductase (Nor), which are classified based on the nature of their electron donor as cNor, qNor or qCuANor (reviewed in [4,9,10,12]). The final step in denitrification consists of the two-electron reduction of nitrous oxide to dinitrogen gas. This reaction is performed by nitrous oxide reductase (Nos), a copper-containing homodimeric soluble protein located in the periplasmic space (reviewed in [9-11,13-15]). Bacteria of the order *Rhizobiales*, collectively referred to as rhizobia, are best known for their ability to establish N_2_-fixing symbiosis on legume roots and on the stems of some aquatic leguminous plants. In addition to fixing N_2_, many rhizobia species have enzyme-encoding genes for some or all of the four reductase reactions in denitrification. Several studies have reported that legume crops contribute to N_2_O production by providing N-rich residues for decomposition [16] and by associating with some rhizobia that are able to denitrify under free-living and under symbiotic conditions, producing N_2_O [17-19]. However, soybean endosymbiont *Bradyrhizobium japonicum* is the only rhizobia species for which it has been demonstrated that the *napEDABC*, *nirK*, *norCBQD* and *nosRZDYFLX* genes are involved in complete denitrification [17,19,20].

*Ensifer* (formerly *Sinorhizobium*) *meliloti* is a rhizobial species that establishes symbiotic N_2_-fixing associations with plants of the genera *Medicago*, *Melilotus* and *Trigonella*. Genes for the complete denitrification pathway are present in the *E. meliloti* pSymA megaplasmid [21,22]. Transcriptomic analyses have shown that the *E. meliloti nap, nir, nor* and *nos* genes are induced in response to O_2_ limitation [23]. Under these conditions, the expression of denitrification genes is coordinated via a two-component regulatory system, FixLJ, and via a transcriptional regulator, FixK [24]. Recent transcriptomic studies demonstrated that denitrification genes (*nirK* and *norC*) and other genes related to denitrification (*azu1*, *hemN*, *nnrU* and *nnrS*) are also induced in response to NO and that the regulatory protein NnrR is involved in the control of this process [25]. In symbiotic association with *M. truncatula* plants*,* recent findings have demonstrated that the *E. meliloti napA* and *nirK* denitrification genes contribute to nitric oxide production in root nodules [26]. Although the regulation and symbiotic characterisation of *E. meliloti* denitrification genes is well understood, the roles of these genes in nitrate reduction through denitrification and in the emission of N_2_O are not known.

Recent results from our group [21] reported the capability of *E. meliloti* to use nitrate or nitrite as respiratory substrates when cells were incubated with an initial oxygen concentration of 2%; however, nitrate and nitrite could not be used as respiratory substrates when the cells were initially incubated anoxically. In the present work, functional analyses of the *E. meliloti napA*, *nirK*, *norC* and *nosZ* genes reveal their involvement in the ability of *E. meliloti* to grow using nitrate as a respiratory substrate and in the expression of denitrification enzymes.

## Results

### Nitrate-dependent growth of *E. meliloti napA*, *nirK*, *norC* and *nosZ* mutants

**Figure 1:**
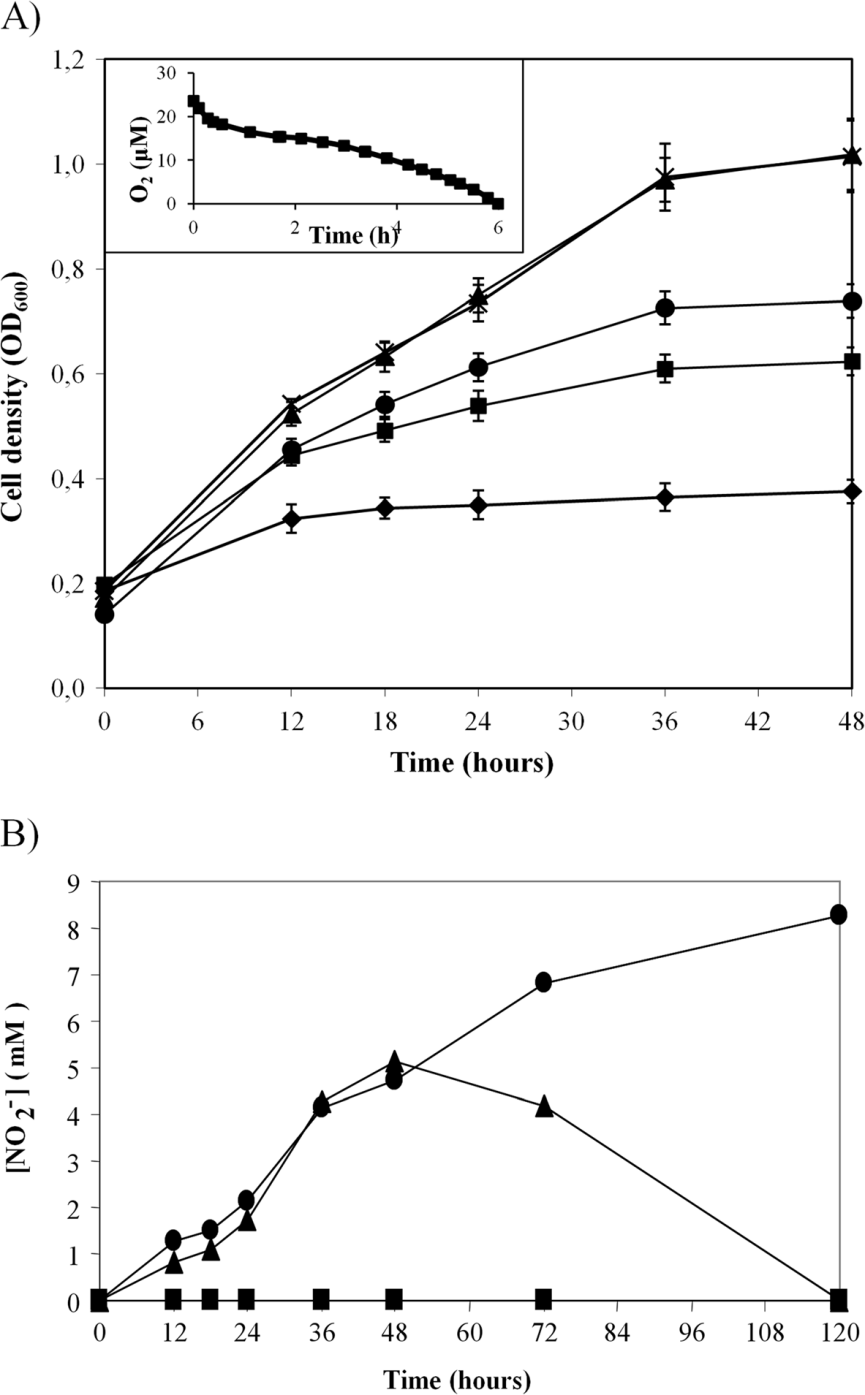
**Growth of *E. meliloti *strains with nitrate. (A)** Growth of *E. meliloti* 1021 (▲) and the *napA* (■), *nirK* (●), *norC* (♦) and *nosZ* (*) mutant strains in MMN under 2% initial O_2_ conditions. The oxygen consumption by the WT cells is also shown (insert). **(B)** The extracellular nitrite concentrations of *E. meliloti* 1021 (▲), *napA* (■) and *nirK* (●) mutant strains. Representative curves of three independent experiments run in triplicate are shown.

To investigate the involvement of denitrification genes in the ability of *E. meliloti* to grow using nitrate as an electron acceptor, the wild-type strains 1021 and 2011 and *napA*, *nirK*, *norC* and *nosZ* mutant strains (Table 1) were incubated in minimal medium (MM) supplemented with 10 mM KNO_3_ (MMN) with an initial O_2_ concentration of 2%, and the growth was determined by monitoring the optical density at 600 nm (OD_600_) (Figure 1). Under these conditions, *E. meliloti* 1021 cells consumed the oxygen present in the atmosphere after incubation for 6 h and reached anoxic conditions (Figure 1A, insert). Similar oxygen consumption rates were observed for strain 2011 and the *napA*, *nirK*, *norC* and *nosZ* mutants (data not shown). Confirming the previous results [21], *E. meliloti* 1021 exhibited a cell density of approximately 1 after 48 h of incubation in MMN (Figure 1A). A similar growth rate was observed after incubation of the wild-type strain 2011 (data not shown). As shown in Figure 1A, the *napA*, *nirK* and *norC* mutant strains exhibited growth defects compared with the WT cells, reaching a turbidity of approximately 0.6, 0.7 and 0.35, respectively, after incubation in MMN for 48 h (Figure 1A). *E. meliloti nosZ* mutant cells demonstrated similar growth to WT cells (Figure 1A), suggesting that *nosZ* was not essential for growth under these conditions. As previously reported for *E. meliloti* 1021 [21], none of the *E. meliloti* denitrification mutants were able to grow in MMN when they were subjected to anoxic conditions starting at the beginning of the incubation period (data not shown). As shown in Figure 1B, after incubation in MMN with an initial O_2_ concentration of 2%, nitrite was not observed in the growth medium of *napA*. However, in the *nirK* mutant, the nitrite concentration increased over the course of the incubation period, reaching a final concentration of 8.3 mM. The WT strains demonstrated a similar rate of nitrite accumulation during the first 48 h; however, this nitrite was depleted over the subsequent 70 h of incubation (Figure 1B).

**Table 1:** Bacterial strains

**Strain**	**Relevant characteristics**	**Reference**
***Ensifer meliloti***		
1021	Wild type; Sm^r^	Meade *et al.,* 1982 [27]
2011	Wild type	Casse *et al*., 1979 [28]
2011mTn5STM.3.02.F08	*napA*::mini-Tn5 Sm^r^, Km^r^	Pobigaylo *et al.,* 2006 [29]
2011mTn5STM.3.13.D09	*napC*::mini-Tn5; Sm^r^, Km^r^	Pobigaylo *et al.,*[29]
2011mTn5STM.1.13.B08	*nirK*::mini-Tn5; Sm^r^, Km^r^	Pobigaylo *et al.,*[29]
SmPl.1021.G1PELR32E8	*norC*::Pl.G1PELR32E8; Sm^r^, Km^r^	Becker *et al.,* 2009 [30]
2011mTn5STM.5.07.B03	*nosZ*::mini-Tn5; Sm^r^, Km^r^	Pobigaylo *et al.*, [29]

### *E. meliloti napA*, *nirK*, *norC* and *nosZ* genes encode functional reductases

The functions of the *E. meliloti* denitrification genes were also investigated by analysing the activities of the denitrification enzymes in WT and *napA*, *nirK*, *norC* and *nosZ* mutants incubated under oxygen-limiting conditions. Cells of the *napA* mutant demonstrated an approximately 11-fold decrease in methyl viologen-dependent nitrate reductase (MV^+^-NR) activity compared with the WT cells after incubation for 18 h in MMN with an initial O_2_ concentration of 2% (Table 2). As observed for the NR activity in *napA* cells, the methyl viologen-dependent nitrite reductase (MV^+^-Nir) activity levels in the *nirK* mutant cells were 10-fold lower than the levels detected in the parental strain when the cells were incubated in MMN with an initial O_2_ concentration of 2% (Table 2). As shown in Table 2, the MV^+^-NR and MV^+^-Nir activities were detected in WT cells incubated under anoxic conditions from the start of the incubation period. Under these conditions, the NR activity levels in *napA* cells and the Nir activity levels in *nirK* cells were undetectable (Table 2).

**Table 2:** **The methyl viologen-dependent (MV**^**+**^**) nitrate reductase (MV**^**+**^**-NR), nitrite reductase (MV**^**+**^**-Nir) and nitric oxide reductase (Nor) activities of *E. meliloti *** **1021 (WT) and the *napA, nirK*, and *norC *** **mutant strains incubated in MMN under 2% initial O**_**2 **_**or anoxic conditions**

**Strain**	**Genotype**	**Oxygen conditions**					
		**2% O**_**2**_			**Anoxia**		
		**MV**^**+**^**-NR**^**a**^	**MV**^**+**^**-NiR**^**b**^	**Nor**^**c**^	**MV**^**+**^**-NR**	**MV**^**+**^**-NiR**	**Nor**
1021	WT	210.93 (10.33)	32.57 (1.42)	563.33 (21.81)	62.96 (5.70)	10.522 (1.465)	335.88 (32.12)
STM.3.02.F08	*napA*	18.86 (3.79)	-	-	n.d.	-	-
STM.1.13.B08	*nirK*	-	3.34 (0.26)	528.26 (20.86)	-	n.d.	308.19 (23.18)
G1PELR32E8	*norC*	-	-	1.11 (0.01)	-	-	2.84 (0.78)

^a^MV^+^-NR and ^b^MV^+^-Nir activities are expressed as nmol NO_2_^-^ produced or consumed · mg protein^-1^ · min^-1^. Nor activity is expressed as nmol NO consumed · mg protein^-1^ · min^-1^. All of the activities were determined after incubation for 18 h. The data are expressed as the means with the standard error in parentheses from at least two different cultures assayed in triplicate. -, not determined; n.d., not detectable.

We also investigated the ability of the *E. meliloti nirK* and *norC* mutants to produce nitric oxide. After incubation for 18 h with an initial O_2_ concentration of 2%, NO production rates were determined in an NO-electrode chamber after adding nitrite to the reaction mixture. A significant decrease in NO production was observed in the *nirK* mutant compared with the WT strain (0.57 ± 0.19 *vs.* 202 ± 15 nmol NO · mg protein^-1^ · min^-1^, respectively), whereas the *norC* mutant produced 4.6-fold more NO than the WT cells (943 ± 4.52 *vs.* 202 ± 15 nmol NO · mg protein^-1^ · min^-1^, respectively). The high levels of NO produced by the *norC* mutant are most likely due to its defect in NO consumption activity. After 18 h of incubation in MMN under an initial O_2_ concentration of 2%, the *norC* mutant cells demonstrated NO consumption activity that was practically abolished compared with the activity of WT cells (Table 2); the same results were observed when the *norC* mutant cells were incubated under initially anoxic conditions.

**Figure 2:**
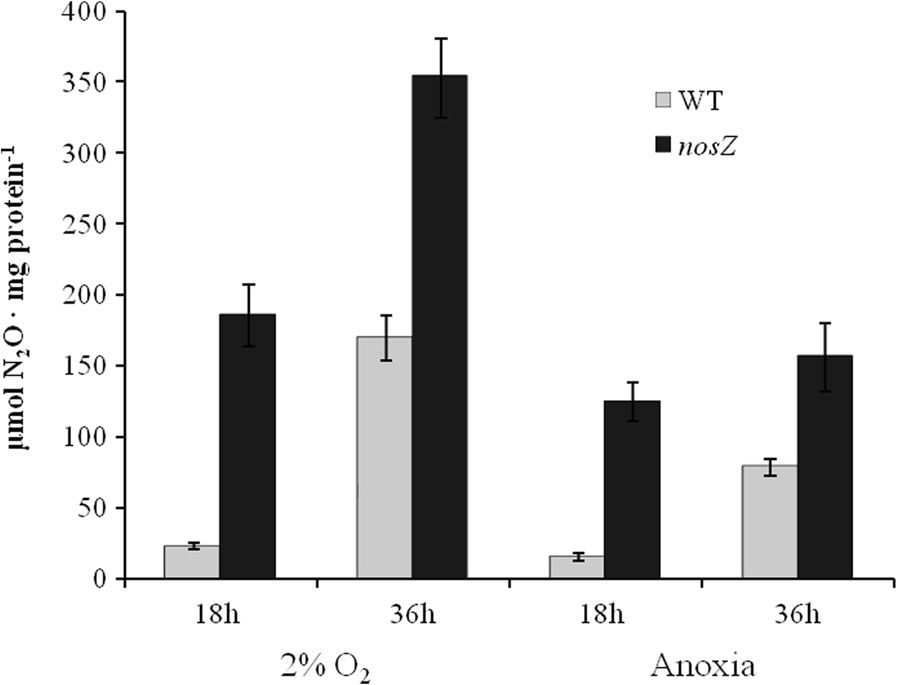
**N**_**2**_**O accumulation in *E. meliloti *1021 (WT) and the *nosZ *mutant incubated in MMN under 2% initial O**_**2 **_**or anoxic conditions.** N_2_O was measured in the headspace of the cultures after 18 and 36 h of incubation. The data represent the means with the standard deviations from at least two different cultures assayed in triplicate.

Figure 2 shows that *E. meliloti* 1021 is able to produce N_2_O after incubation in MMN under an initial O_2_ concentration of 2% and under anoxic conditions. Under both conditions, the *nosZ* mutant cells achieved N_2_O accumulation values of approximately 8- and 2-fold higher than the values produced by WT cells after 18 h and 36 h of incubation in MMN, respectively (Figure 2).

### Identification of *E. meliloti* NorC

**Figure 3:**
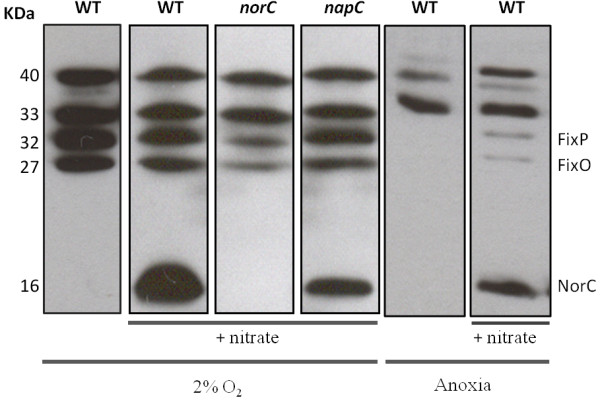
**Haem-stained proteins of membranes prepared from *E. meliloti *1021 (WT) and the *norC *and *napC *mutants incubated in MM or MMN for 24 h under 2% initial O**_**2 **_**or anoxic conditions.** Each lane contains 25 μg of membrane proteins. Haem-stained *c*-type cytochromes identified previously (FixP and FixO) and in this work (NorC) are specified in the right margin. Apparent protein molecular masses (kDa) are shown in the left margin.

As previously reported by Torres and colleagues [31], four haem-stained bands of 40, 33, 32 and 27 kDa were detected in *E. meliloti* 1021 cells grown in minimal media (MM) with an initial O_2_ concentration of 2% in the headspace (Figure 3, lane 1). Although the identities of the 40 kDa and 33 kDa proteins are unknown, the 32 kDa and 27 kDa *c*-type cytochromes were identified as the *E. meliloti* FixP and FixO proteins, respectively, which are subunits of the *cbb*_3_-type high-affinity cytochrome *c* oxidase encoded by the *fixNOQP* operon [31]. The addition of nitrate to the growth medium revealed a haem-stainable band of approximately 16 kDa in the membranes of the WT cells (Figure 3, lane 2). This protein was absent in the *norC* mutant when it was incubated with a 2% initial oxygen concentration in MMN (Figure 3, lane 3), which identifies this *c*-type cytochrome as the NorC component of the *E. meliloti* 1021 nitric oxide reductase. As shown in Figure 3 (lane 4), membranes from the *napC* mutant presented a similar band pattern to that of membranes from the WT cells incubated under an initial O_2_ concentration of 2% with nitrate (Figure 3, lanes 2 and 4). These results did not permit us to identify the *E. meliloti* NapC protein, which has a predicted size of 25 kDa. In contrast, in other rhizobia species, such as *B. japonicum*, NapC has been detected via haem-staining analyses and identified as a protein approximately 25 kDa in size [32].

**Figure 4:**
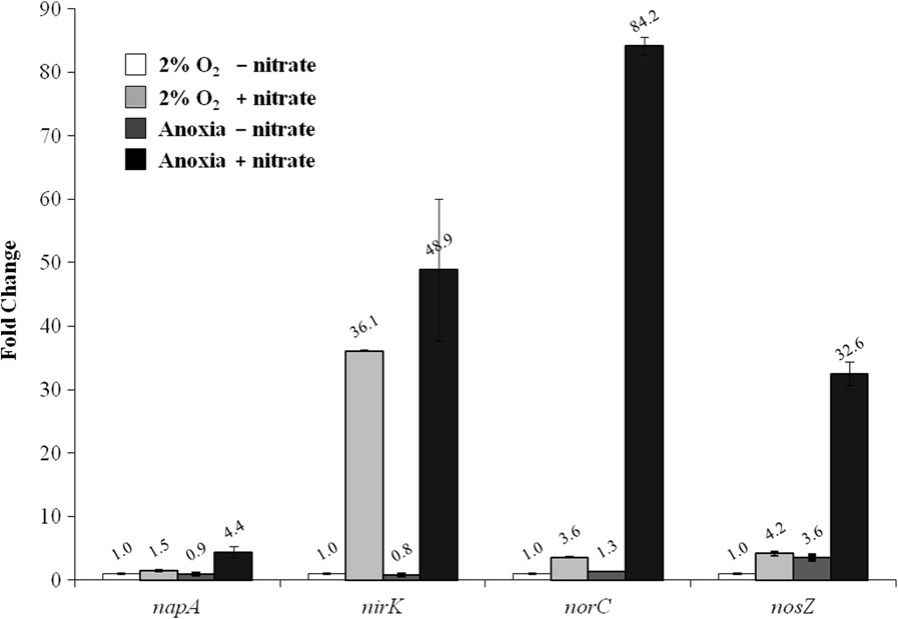
**Expression of *E. meliloti *1021 *napA *, *nirK*, *norC *and *nosZ *denitrification genes in cells incubated for 12 h in MM or MMN under an initial oxygen concentration of 2% or under anoxic conditions.** The transcription levels were quantified using qRT-PCR with total RNA samples as the templates. The data were analysed using the standard curve method (*nirK* data were analysed with the comparative C_T_ method), and the expression levels were normalised against the *E. meliloti smc00128* gene as an internal standard. The values expressed relative to the values of cells incubated under 2% initial O_2_ in the absence of nitrate are the means and standard deviations of three independent experiments run in triplicate.

When the cells were subjected to anoxic conditions starting at the beginning of the incubation period, a strong defect in FixP and FixO expression was observed compared with the expression levels detected in cells incubated with an initial O_2_ concentration of 2% (Figure 3, lanes 1 and 5). Only proteins approximately 40 and 33 kDa in size could be detected in the anoxically incubated cells. These 40 kDa and 33 kDa proteins were also present in cells grown under oxic conditions [31]. These proteins might remain in the membranes of cells that are grown aerobically prior to the anoxic incubation period. As shown in Figure 3 (lanes 2 and 6), nitrate-dependent NorC expression decreased under anoxic conditions compared with cells incubated with an initial O_2_ concentration of 2%. As observed for NorC, the expression of FixP and FixO was weak in the membranes from the anoxically incubated cells in the presence of nitrate (Figure 4, lanes 2 and 6).

### Expression of *E. meliloti* denitrification genes

We analysed the expression of the *E. meliloti napA*, *nirK*, *norC* and *nosZ* genes using qRT-PCR analyses. With the exception of *nirK* expression, which was induced 36-fold by nitrate, the presence of nitrate in the growth medium of cells incubated under an initial O_2_ concentration of 2% provoked the induction of *napA*, *norC* and *nosZ* expression by 1.5-, 3.6- and 4.2-fold, respectively, compared with the expression observed in the absence of nitrate (Figure 4). When the cells were incubated anoxically from the beginning of culture, the *napA, nirK, norC* and *nosZ* genes were induced approximately 4-, 48-, 84- and 32-fold by nitrate compared with the expression levels observed after a 12 h incubation in MM at an initial O_2_ concentration of 2% (Figure 4). These results indicate that the maximal expression of the *E. meliloti napA*, *nirK*, *norC* and *nosZ* denitrification genes occurs when the cells are initially incubated anoxically and when nitrate is present in the growth medium.

## Discussion

*E. meliloti* has been considered a partial denitrifier because of its traditionally reported inability to use nitrate as an electron acceptor for ATP generation and growth under anoxic conditions [18,33]. Recent results from our group confirmed the inability of *E. meliloti* to grow via nitrate respiration when cells were initially incubated under anoxic conditions [21]; however, *E. meliloti* 1021 was able to use nitrate as a respiratory substrate when cells were initially incubated with 2% O_2_ in the headspace [21]. Under these conditions, O_2_ was consumed after 6 h of incubation, as we demonstrated in the present manuscript. In this work, we demonstrated that *E. meliloti nap* genes are involved in *E. meliloti* nitrate-dependent growth and that nitrite derived from nitrate reduction is produced by Nap. The low levels of NR activity observed in the *napA* mutant explain the growth defect and the inability of this strain to produce nitrite in cells incubated in MMN with 2% initial O_2_. The majority of the most well-characterised denitrifying bacteria use the membrane-bound nitrate reductase (Nar) to catalyse the first step of denitrification. In contrast to Nar, which has a respiratory function, Nap systems demonstrate a range of physiological functions, including the disposal of reducing equivalents during aerobic growth on reduced carbon substrates or anaerobic nitrate respiration [2-6]. Our results support the proposed role of Nap in nitrate respiration. Some rhizobial species, such as *Pseudomonas* sp. G179 (*Rhizobium galegae*) and *Bradyrhizobium japonicum*, could express *nap* genes under anaerobic conditions, and the disruption of these genes is lethal for growth under denitrifying conditions [32,34].

Whereas the deletion of *nosZ* did not have a significant effect on the ability of *E. meliloti* to respire nitrate and increase growth yield, the *nirK* and *norC* mutants exhibited clear defects in nitrate-dependent growth, most likely because of the toxicity of the intermediates nitrite and nitric oxide, respectively. Nitrite or NO were accumulated by the *nirK* and *norC* mutants, respectively, because of the strong defects in Nir and Nor activities observed in these mutants compared with WT levels. Similar phenotypes for *nirK* and *norC* mutants were reported for *B. japonicum*[35,36] and *Rhizobium etli*[37]. The increased levels of N_2_O accumulated by the *nosZ* mutant relative to the WT cells indicated that this gene is involved in nitrous oxide reduction in *E. meliloti*. Similar observations were noted with a *B. japonicum nosZ* mutant [38]. In addition to demonstrate the involvement of the *E. meliloti napA*, *nirK*, *norC* and *nosZ* genes in nitrate, nitrite, nitric oxide and nitrous oxide reduction, respectively, we have identified the NorC subunit of nitric oxide reductase as a cytochrome c that is approximately 16 kDa in size.

Growth experiments in this study and in previous studies [21] clearly demonstrated that *E. meliloti* utilises nitrate-dependent growth when transitioning to anoxic conditions occurs when cells are incubated under an initial O_2_ concentration of 2%; however, nitrate-dependent growth does not occur when cells are subjected to anoxic conditions starting at the beginning of the incubation period. To understand the differential responses of *E. meliloti* denitrification capability to these different anoxically induced conditions, we investigated the ability of *E. meliloti* to express the denitrification genes in cells incubated under 2% initial O_2_ compared with cells initially subjected to anoxic conditions. Despite the inability of *E. meliloti* to grow, we demonstrated that the *napA*, *nirK, norC* and *nosZ* denitrification genes were fully induced in cells initially subjected to anoxia and in the presence of nitrate. Furthermore, denitrification enzymes are active in cells initially incubated under anoxic conditions; we were able to detect significant MV^+^-NR, MV^+^-NiR and Nor activity levels and N_2_O production under these conditions. In contrast, the protein levels corresponding to NorC and the FixP and FixO components of the high affinity *cbb*_3_ oxidase were very weak after incubation of the cells under anoxic conditions starting at the beginning of the incubation period. The latter observations might explain the limited nitrate-dependent growth capacity of *E. meliloti* when anoxic conditions are induced starting at the beginning of the growth period. Under these conditions, cells would be trapped, without energy, and they would be unable to produce the proteins required to cope with the oxygen-limiting conditions, most likely because of the lack of energy. Supporting this hypothesis, it was reported in *Pseudomonas* sp. G59 that the formation of nitrate reductase and nitrous oxide reductase did not occur under aerobic or anaerobic conditions; however, nitrate reductase and nitrous oxide reductase were produced under microaerobic incubation [39]. The latter study suggests that dependence on microaerobiosis for the formation of these reductases was attributable to an inability to produce energy anaerobically until these anaerobic respiratory enzymes formed [39]. Recent studies have shown that the soil bacterium *Agrobacterium tumefaciens* is unable to maintain balanced expression of denitrification genes if oxygen depletion occurs too quickly [40,41]. Similarly, the soil bacterium *P. denitrificans* appears unable to effectively switch from oxic to anoxic respiration, leaving a large fraction of the cell population in anoxia without a chance to express the denitrification proteome [41]. As suggested by Nadeem and co-workers [42], “microaerobic” denitrification is an essential trait for securing an efficient transition to anaerobic denitrification. Considering that *B. japonicum,* which is able to grow under anoxic nitrate-respiring conditions, is a slow-growth bacterium and *E. meliloti* is a fast-growth bacterium, the transition from oxic to anoxic metabolism might be different in these species. Supporting this suggestion, we observed that *B. japonicum* cells are able to express the FixO and FixP subunits of the *cbb*_3_ oxidase under anoxic conditions (E. Bueno, personal communication). However, as shown in this work, *E. meliloti* does not express the FixO and FixP proteins under anoxic conditions. A lack of the energy necessary for protein synthesis might contribute to the inability of *E. meliloti* to grow via nitrate respiration when cells are initially incubated anoxically.

## Conclusion

The potential impact of denitrification by plant endosymbiotic bacteria on the emission of the greenhouse gas N_2_O has been poorly investigated. The results of this work demonstrate the involvement of the *napA*, *nirK*, *norC* and *nosZ* genes in the previously reported ability of *E. meliloti* to grow via nitrate respiration when cells are subjected initially to 2% O_2_. Furthermore, the roles of the reductases encoded by *napA*, *nirK*, *norC* and *nosZ* in nitrite, nitric oxide, N_2_O production and N_2_O reduction, respectively, were demonstrated. Thus, our results contribute to the investigation of the unexplored genetic basis for denitrification in the alfalfa endosymbiont *E. meliloti*. This knowledge will be instrumental in the development of agricultural strategies and management practices for mitigating the release of N_2_O from legume crops.

## Methods

### Bacterial strains and growth conditions

The bacterial strains used in this study are listed in Table 1. *E. meliloti* strains were routinely grown aerobically at 30°C in tryptone yeast (TY) complete medium [43]. These cultures were then used as the inocula for subsequent incubation experiments, which were performed in minimal medium (MM) [44] or in MM medium supplemented with 10 mM KNO_3_ (MMN); the cells were subjected to two experimental oxygen-limiting conditions. In the first set of experiments, 17-ml serum tubes or 500-ml flasks containing 5 or 200 ml medium, respectively, were sealed with rubber septa, and the headspace atmospheres were replaced with a gas mixture (2% oxygen, 98% argon) at the starting point of the incubation. In the second experiment, the cells were incubated in completely filled 200-ml bottles or 17-ml tubes without added oxygen; these conditions are referred to throughout the manuscript as “anoxic conditions”. Antibiotics were added to the cultures at the following concentrations (μg · ml^-1^): streptomycin, 200; and kanamycin, 200.

### Headspace O_2_ measurements

After inoculation at an OD_600_ of 0.2, 1 ml of each culture was placed in a 3-ml thermostatted and magnetically stirred reaction chamber with an O_2_ electrode (Hansatech, Norkfolk, England). The headspace atmosphere in the chamber was replaced with a gas mixture (2% oxygen, 98% argon) at the starting point of the incubation. The kinetics of oxygen depletion in the chamber were monitored.

### Determination of nitrate reductase and nitrite reductase activity

*E. meliloti* cells were incubated (initial OD_600_ of approximately 0.15-0.2) under 2% initial oxygen or under anoxic conditions for 18 h in MMN medium. The cells were harvested by centrifugation at 8000 *g* for 10 min at 4°C, washed with 50 mM Tris/HCl buffer (pH 7.5) until no nitrite was detected and then resuspended in 0.5 ml of the same buffer. The methyl viologen-dependent nitrate reductase (MV^+^-NR) activity was analysed essentially as described by Delgado and colleagues (2003) [32]. To determine the methyl viologen-dependent nitrite reductase (MV^+^-Nir) activity, the reaction mixture contained 50 mM Tris/HCl buffer (pH 7.5), 200 μM NaNO_2_, 400 μM methyl viologen (MV) and 100 μl of cell suspension (0.02–0.04 mg of protein). The reaction was started by the addition of 50 μl of freshly prepared sodium dithionite solution (30 mg · ml^-1^ in 300 mM NaHCO_3_). After incubation for 20 min at 30°C, the reaction was stopped by vigorous shaking until the samples lost their blue colour.

### Haem-staining analysis

*E. meliloti* cells grown aerobically in 150 ml of TY medium were harvested by centrifugation at 8000 *g* for 5 min, washed twice with MM, resuspended in 200 ml of MM or MMN at an OD_600_ of 0.15-0.2 and incubated under 2% initial O_2_ or anoxic (filled bottles) conditions for 24 h. The cell pellets were resuspended in 3 ml of 50 mM potassium phosphate buffer (pH 7) containing 100 μM 4-(2-aminoethyl) benzene-sulfonyl fluoride hydrochloride (ABSF), RNAse (20 μg · ml^-1^) and DNAse I (20 μg · ml^-1^). The cells were disrupted using a French pressure cell at a constant pressure of approximately 1000 psi (SLM Aminco, Jessup, MD, USA). The cell extract was centrifuged at 10,000 *g* for 20 min to remove the unbroken cells, and the supernatant was centrifuged at 140,000 *g* for 1 h. The membrane pellet was resuspended in 100 μl of the same buffer. The membrane protein aliquots were diluted in sample buffer [124 mM Tris–HCl, pH 7.0, 20% glycerol, 4.6% sodium dodecyl sulphate (SDS) and 50 mM 2-mercaptoethanol] and incubated at room temperature for 10 min. The membrane proteins were separated at 4°C using 12% SDS-polyacrylamide gel electrophoresis, transferred to a nitrocellulose membrane and stained for haem-dependent peroxidase activity, as described previously [45], using the SuperSignal chemiluminescence detection kit (Pierce, Thermo Fisher Scientific, IL, USA).

### Analytical methods

The nitrite concentration was estimated after diazotisation by adding the sulphanilamide/naphthylethylene diamine dihydrochloride reagent [46]. The protein concentration was estimated using the Bradford method (Bio-Rad Laboratories, Richmond, CA) with a standard curve constructed with varying bovine serum albumin concentrations.

### Nitric oxide determination

*E. meliloti* cells were incubated at an OD_600_ of 0.15-0.2 in MMN under 2% initial O_2_ or anoxic conditions, harvested and washed similar to the NR or Nir activity assays. Nitric oxide was measured amperometrically with a 2-mm ISONOP electrode APOLO 4000® (World Precision Inst., Sarasota, FL, USA) in a 3-ml thermostatted and magnetically stirred reaction chamber [47]. The membrane-covered electrode was situated at the bottom of the chamber above the stirrer, and the reactants were injected using a Hamilton syringe through a port in the glass stopper. To determine the net production of NO, the 3-ml cuvette was filled with 1.410 ml of 25 mM phosphate buffer (pH 7.4), 250 μl (0.1-0.2 mg protein) of a cellular solution, 100 μl of an enzymatic mix containing glucose oxidase (*Aspergillus niger*) (80 units/2 ml) and catalase (bovine liver) (500 units/2 ml), 90 μl of 1 M sodium succinate and 100 μl of 320 mM glucose. When oxygen was consumed and a steady base line was observed, 50 μl of 1 M NaNO_2_ was added to the cuvette to begin the reaction. Each assay was continued until NO was detected. To determine the NO consumption rates, the electrode chamber was filled with 1.655 ml of 25 mM phosphate buffer (pH 7.4), 5 μl (0.02-0.04 mg protein) of a cellular solution, 100 μl of an enzymatic mix containing glucose oxidase (*Aspergillus niger*) (80 units/2 ml) and catalase (bovine liver) (500 units/2 ml), 90 μl of 1 M sodium succinate and 100 μl of 320 mM glucose. Once a steady base line was observed, 50 μl of a saturated NO solution (1.91 mM at 20°C) was added to the cuvette to start the reaction. Each assay was continued until NO detection dropped to zero (when all of the NO was consumed).

### Nitrous oxide determination

*E. meliloti* cells were incubated in MMN with an initial O_2_ concentration of 2% in the headspace or anoxically. After 18 or 36 h of incubation, 500-μl gaseous aliquots were taken from the culture headspaces to determine the N_2_O level. In anoxic cultures (filled tubes), headspace was created by transferring 10 ml of liquid culture into a 20-ml headspace vial (Supelco®). Gas–liquid phase equilibration was performed by incubating the vials for 2 h at 30°C and at 185 rpm. To stop cell growth, 200 μl of 1 mg · ml^-1^ HgCl_2_ was added to each vial. The N_2_O production in liquid cultures was corrected using the dissolved N_2_O Bunsen solubility coefficient (47.2% at 30°C). Then, N_2_O was measured with a gas chromatograph type HP 4890D equipped with an electron capture detector (ECD). The column was packed with Porapak Q 80/100 MESH (6 ft), and the carrier gas was N_2_ at a flow rate of 23 ml/min. The injector, column and detector temperatures were 125, 60 and 375°C, respectively. The N_2_O peaks were integrated using GC ChemStation Software (Agilent Technologies^©^ 1990–2003). The samples were injected manually through a Hamilton® Gastight syringe. The concentrations of N_2_O in each sample were calculated from pure nitrous oxide standards (Air Liquid, France).

### Quantitative real-time PCR analysis

For immediate stabilisation of the bacterial RNA, the RNAprotect Bacteria Reagent (Qiagen Valencia, CA, USA) was added directly to cells incubated for 12 h in MM or MMN with an initial headspace O_2_ concentration of 2% or anoxically. Bacterial lysis was performed by resuspension and incubation of the cell pellet in 1 mg/ml lysozyme from chicken egg whites (Sigma-Aldrich) in Tris-EDTA buffer, pH 8.0. The total RNA was isolated using the RNeasy Mini kit (Qiagen). The isolated RNA was subjected to DNase (Qiagen) treatment. The RNA was quantified using a NanoDrop 1000 Spectrophotometer (Thermo Scientific, USA), and intactness was verified by the visual inspection of rRNA bands in electrophoretically separated total RNA [48]. Reverse transcription reactions were performed with 0.8 μg of total RNA per reaction using the First Strand cDNA Synthesis kit for RT-PCR (Roche) with random hexamers. The cDNA synthesis reaction mixture was diluted 50 times with distilled water before use in real-time PCR analysis.

The primers for the PCR reactions were designed using Primer Express v3.0 software (PE Applied Biosystems, Foster City, CA, USA) to have a melting temperature of approximately 57°C to 62°C and to produce a PCR product of approximately 50 to 100 bp. The primer sequences were as follows: *napA* (forward, 5′-CCGGCTATCGTGGCAAGA-3′; reverse, 5′-CGGGAAGCTGTCGACATTG-3′); *nirK* (forward, 5′-CCGCGCGACGCAAA-3′; reverse, 5′-TCGAGCGTATCGGCATAGG-3′); *norC* (forward, 5′-AGCTCACAGAGCAGGAACTGAAC-3′; reverse, 5′-TGATGCGGCTCGTCCATT-3′); and *nosZ* (forward, 5′-CGAGGATCTCACGCATGGAT-3′; reverse, 5′-GCGGTGCAACCTCCATGT-3′). *sMC00128* was used as an internal standard [49,50] (forward, 5′-ACGAGATCGAGATCGCCATT-3′; reverse, 5′-CGAACGAGGTCTTCAGCATGA-3′).

Each PCR reaction contained 7.5 μl of SYBR Green PCR master mix (PE Applied Biosystems), 5 μl of cDNA and various final concentrations of each primer depending on the studied gene. This concentration was 0.2 μM for *norC* and *sMC00128* and 0.4 μM for *napA*, *nosZ* and *nirK*. The final volume of the PCR reactions was 15 μl. The real-time PCR reactions were performed on a 7300 Real Time PCR System (PE Applied Biosystems). The initial denaturing time of 10 min was followed by 40 PCR cycles consisting of 95°C for 15 s and 60°C for 60 s. A melting curve was run after the PCR cycles. During real-time PCR, the efficiency of *nirK* gene amplification was approximately equal to that of the housekeeping (internal standard) gene; in this case, the comparative C_T_ method (also called ∆∆C_T_ method) was applied for relative quantification. For the other genes, the amplification efficiencies were different from that of the housekeeping gene; the comparative C_T_ method could not be applied, and it was necessary to use the standard curve method. The data were analysed using the 7300 System Software (PE Applied Biosystems). The gene expression values under different conditions were expressed relative to the values of cells incubated under an initial O_2_ concentration of 2% in the absence of nitrate.

## Competing interests

The authors declare that they have no competing interests.

## Authors’ contributions

MJT and MJD conceived of the study. MJT and MIR carried out the phenotypic analyses of the *E. meliloti* denitrification mutants. TC and JJP participated in the gene expression experiments. MJD and EJB supported the research. MJT and MJD wrote the manuscript. EJB coordinated and critically revised the manuscript. All of the authors read and approved the manuscript.

## References

[B1] Bates BC Kundzewicz ZW Wu S Palutikof JP Climate Change and Water.Technical Paper of the Intergovernmental Panel on Climate Change 2008 Geneva, Switzerland: IPCC Secretariat 210

[B2] Gonzalez PJ Correia C Moura I Brondino CD Moura JJ Bacterial nitrate reductases: molecular and biological aspects of nitrate reduction J Inorg Biochem 2006 100 5–6 1015 1023 16412515 10.1016/j.jinorgbio.2005.11.024

[B3] Kraft B Strous M Tegetmeyer HE Microbial nitrate respiration–genes, enzymes and environmental distribution J Biotechnol 2011 155 1 104 117 10.1016/j.jbiotec.2010.12.025 21219945

[B4] Richardson DJ Moir JWB Redox complexes of the nitrogen cycle Nitrogen Cycling in Bacteria 2011 Norkfolk, UK: Caister Academic Press 23 39

[B5] Richardson DJ Berks BC Russell DA Spiro S Taylor CJ Functional, biochemical and genetic diversity of prokaryotic nitrate reductases Cell Mol Life Sci 2001 58 2 165 178 10.1007/PL00000845 11289299 PMC11146511

[B6] Richardson DJ van Spanning RJ Ferguson SJ Bothe H, Ferguson SJ, Newton WE The prokaryotic nitrate reductases Biology of the Nitrogen Cycle 2007 The Nerthelands: Elservier 21 35

[B7] Rinaldo S Arcovito A Giardina G Castiglione N Brunori M Cutruzzola F New insights into the activity of *Pseudomonas aeruginosa* cd1 nitrite reductase Biochem Soc Trans 2008 36 Pt 6 1155 1159 19021515 10.1042/BST0361155

[B8] Rinaldo S Cutruzzola F Bothe H, Ferguson SJ, Newton WE Nitrite reductases in denitrification Biology of the Nitrogen Cycle 2007 The Netherlands: Elservier 37 56

[B9] van Spanning RJ Delgado MJ Richardson DJ Werner D, Newton WE The nitrogen cycle: denitrification and its relationship to N_2_ fixation Nitrogen Fixation in Agriculture, Forestry, Ecology and the Environment 2005 Netherlands: Springer 277 342

[B10] van Spanning RJ Richardson DJ Ferguson SJ Bothe H, Ferguson SJ, Newton WE Introduction to the biochemistry and molecular biology of denitrification Biology of the Nitrogen Cycle.3-20 2007 Amsterdam: Elsevier Science

[B11] van Spanning RJ Moir JWB Structure, function, regulation and evolution of the nitrite and nitrous oxide reductase: denitrification enzymes with a b-propeller fold Nitrogen Cycling in Bacteria 2011 Norkfolk, UK: Caister Academic Press 135 161

[B12] de Vries R Suharti R Pouvreau LAM Bothe H, Ferguson SJ, Newton WE Nitric oxide reductase: structural variations and catalytic mechanism Biology of the Nitrogen Cycle 2007 The Netherlands: Elsevier 57 66

[B13] Zumft WG Kroneck PM Respiratory transformation of nitrous oxide (N_2_O) to dinitrogen by Bacteria and Archaea Adv Microb Physiol 2007 52 107 227 17027372 10.1016/S0065-2911(06)52003-X

[B14] Thomson AJ Giannopoulos G Pretty J Baggs EM Richardson DJ Biological sources and sinks of nitrous oxide and strategies to mitigate emissions Philos Trans R Soc Lond B Biol Sci 2012 367 1593 1157 1168 10.1098/rstb.2011.0415 22451101 PMC3306631

[B15] Hartsock A Shapleigh JP Identification, functional studies, and genomic comparisons of new members of the NnrR regulon in *Rhodobacter sphaeroides* J Bacteriol 2010 192 4 903 911 10.1128/JB.01026-09 19966004 PMC2812982

[B16] Baggs EM Rees RM Smith KA Vinten AJA Nitrous oxide emission from soils after incorporating crop residues Soil Use Manag 2000 16 2 82 87

[B17] Bedmar EJ Robles EF Delgado MJ The complete denitrification pathway of the symbiotic, nitrogen-fixing bacterium *Bradyrhizobium japonicum* Biochem Soc Trans 2005 33 Pt 1 141 144 15667287 10.1042/BST0330141

[B18] Garcia-Plazaola JI Becerril JM Arrese-Igor C Hernandez A Gonzalez-Murua C Aparicio-Tejo PM Denitrifying ability of thirteen *Rhizobium meliloti* strains Plant Soil 1993 149 43 50 10.1007/BF00010761

[B19] Sánchez C Bedmar EJ Delgado MJ Moir JWB Denitrification in Legume-associated endosymbiotic Bacteria Nitrogen cycling in Bacteria 2011 Norfolk, UK: Caister Academic Press 197 210

[B20] Delgado MJ Casella S Bedmar EJ Bothe H, Ferguson SJ, Newton WE Denitrification in rhizobia-legume symbiosis Biology of the Nitrogen Cycle 2007 Amsterdam: Elsevier Science 83 93

[B21] Torres MJ Rubia MI Bedmar EJ Delgado MJ Denitrification in *Sinorhizobium meliloti* Biochem Soc Trans 2011 39 6 1886 1889 10.1042/BST20110733 22103545

[B22] Barnett MJ Fisher RF Jones T Komp C Abola AP Barloy-Hubler F Bowser L Capela D Galibert F Gouzy J Gurjal M Hong A Huizar L Hyman RW Kahn D Kahn ML Kalman S Keating DH Palm C Peck MC Surzycki R Wells DH Yeh KC Davis RW Federspiel NA Long SR Nucleotide sequence and predicted functions of the entire *Sinorhizobium meliloti* pSymA megaplasmid Proc Natl Acad Sci U S A 2001 98 17 9883 9888 10.1073/pnas.161294798 11481432 PMC55547

[B23] Becker A Berges H Krol E Bruand C Ruberg S Capela D Lauber E Meilhoc E Ampe F de Bruijn FJ Fourment J Francez-Charlot A Kahn D Kuster H Liebe C Puhler A Weidner S Batut J Global changes in gene expression in *Sinorhizobium meliloti* 1021 under microoxic and symbiotic conditions Mol Plant Microbe Interact 2004 17 3 292 303 10.1094/MPMI.2004.17.3.292 15000396

[B24] Bobik C Meilhoc E Batut J FixJ: a major regulator of the oxygen limitation response and late symbiotic functions of *Sinorhizobium meliloti* J Bacteriol 2006 188 13 4890 4902 10.1128/JB.00251-06 16788198 PMC1482993

[B25] Meilhoc E Cam Y Skapski A Bruand C The response to nitric oxide of the nitrogen-fixing symbiont *Sinorhizobium meliloti* Mol Plant Microbe Interact 2010 23 6 748 759 10.1094/MPMI-23-6-0748 20459314

[B26] Horchani F Prevot M Boscari A Evangelisti E Meilhoc E Bruand C Raymond P Boncompagni E Aschi-Smiti S Puppo A Brouquisse R Both plant and bacterial nitrate reductases contribute to nitric oxide production in *Medicago truncatula* nitrogen-fixing nodules Plant Physiol 2011 155 2 1023 1036 10.1104/pp.110.166140 21139086 PMC3032450

[B27] Meade HM Long SR Ruvkun GB Brown SE Ausubel FM Physical and genetic characterization of symbiotic and auxotrophic mutants of *Rhizobium meliloti* induced by transposon Tn*5* mutagenesis J Bacteriol 1982 149 1 114 122 6274841 10.1128/jb.149.1.114-122.1982 PMC216598

[B28] Casse F Boucher C Julliot JS Michel M Dénarié J Identification and Characterization of Large Plasmids in *Rhizobium meliloti* using Agarose Gel Electrophoresis J Gen Microbiol 1979 113 2 229 242 10.1099/00221287-113-2-229

[B29] Pobigaylo N Wetter D Szymczak S Schiller U Kurtz S Meyer F Nattkemper TW Becker A Construction of a large signature-tagged mini-Tn5 transposon library and its application to mutagenesis of *Sinorhizobium meliloti* Appl Environ Microbiol 2006 72 6 4329 4337 10.1128/AEM.03072-05 16751548 PMC1489598

[B30] Becker A Barnett MJ Capela D Dondrup M Kamp PB Krol E Linke B Ruberg S Runte K Schroeder BK Weidner S Yurgel SN Batut J Long SR Puhler A Goesmann A A portal for rhizobial genomes: RhizoGATE integrates a *Sinorhizobium meliloti* genome annotation update with postgenome data J Biotechnol 2009 140 1–2 45 50 19103235 10.1016/j.jbiotec.2008.11.006 PMC2656595

[B31] Torres MJ Hidalgo-Garcia A Bedmar EJ Delgado MJ Functional analysis of the copy 1 of the *fixNOQP* operon of Ensifer meliloti under free-living micro-oxic and symbiotic conditions J Appl Microbiol 2013 114 6 1772 1781 10.1111/jam.12168 23414432

[B32] Delgado MJ Bonnard N Tresierra-Ayala A Bedmar EJ Muller P The *Bradyrhizobium japonicum napEDABC* genes encoding the periplasmic nitrate reductase are essential for nitrate respiration Microbiology 2003 149 Pt 12 3395 3403 14663073 10.1099/mic.0.26620-0

[B33] García-Plazaola JI Denitrification in lucerne nodules is not involved in nitrite detoxification Plant Soil 1996 182 149 155 10.1007/BF00011003

[B34] Bedzyk L Wang T Ye RW The periplasmic nitrate reductase in *Pseudomonas sp*. strain G-179 catalyzes the first step of denitrification J Bacteriol 1999 181 9 2802 2806 10217771 10.1128/jb.181.9.2802-2806.1999 PMC93722

[B35] Velasco L Mesa S Delgado MJ Bedmar EJ Characterization of the *nirK* gene encoding the respiratory, Cu-containing nitrite reductase of *Bradyrhizobium japonicum* Biochim Biophys Acta 2001 1521 1–3 130 134 11690645 10.1016/s0167-4781(01)00279-2

[B36] Mesa S Velasco L Manzanera ME Delgado MJ Bedmar EJ Characterization of the *norCBQD* genes, encoding nitric oxide reductase, in the nitrogen fixing bacterium *Bradyrhizobium japonicum* Microbiology 2002 148 Pt 11 3553 3560 12427946 10.1099/00221287-148-11-3553

[B37] Gomez-Hernandez N Reyes-Gonzalez A Sanchez C Mora Y Delgado MJ Girard L Regulation and symbiotic role of *nirK* and *norC* expression in *Rhizobium etli* Mol Plant Microbe Interact 2011 24 2 233 245 10.1094/MPMI-07-10-0173 21043576

[B38] Velasco L Mesa S Xu CA Delgado MJ Bedmar EJ Molecular characterization of *nosRZDFYLX* genes coding for denitrifying nitrous oxide reductase of *Bradyrhizobium japonicum* Antonie Van Leeuwenhoek 2004 85 3 229 235 15028871 10.1023/B:ANTO.0000020156.42470.db

[B39] Aida T Hata S Kusunoki H Temporary low oxygen conditions for the formation of nitrate reductase and nitrous oxide reductase by denitrifying *Pseudomonas sp*. G59 Can J Microbiol 1986 32 7 543 547 10.1139/m86-101 3742333

[B40] Bergaust L Shapleigh J Frostegard A Bakken L Transcription and activities of NOx reductases in *Agrobacterium tumefaciens*: the influence of nitrate, nitrite and oxygen availability Environ Microbiol 2008 10 11 3070 3081 10.1111/j.1462-2920.2007.01557.x 18312398

[B41] Bergaust L Mao Y Bakken LR Frostegard A Denitrification response patterns during the transition to anoxic respiration and posttranscriptional effects of suboptimal pH on nitrous oxide reductase in *Paracoccus denitrificans* Appl Environ Microbiol 2010 76 19 6387 6396 10.1128/AEM.00608-10 20709842 PMC2950438

[B42] Nadeem S Dorsch P Bakken LR The significance of early accumulation of nanomolar concentrations of NO as an inducer of denitrification FEMS Microbiol Ecol 2013 83 3 672 684 10.1111/1574-6941.12024 23035849

[B43] Beringer JE R factor transfer in *Rhizobium leguminosarum* J Gen Microbiol 1974 84 1 188 198 10.1099/00221287-84-1-188 4612098

[B44] Robertsen BK Aman P Darvill AG McNeil M Albersheim P The structure of acidic extracellular polysaccharides secreted by *Rhizobium leguminosarum and Rhizobium trifolii* Plant Physiol 1981 67 3 389 400 10.1104/pp.67.3.389 16661681 PMC425692

[B45] Vargas C McEwan AG Downie JA Detection of c-type cytochromes using enhanced chemiluminescence Anal Biochem 1993 209 2 323 326 10.1006/abio.1993.1127 8385891

[B46] Nicholas DJD Nason A Colowick SP, Kaplan NO Determination of nitrate and nitrite Methods in Enzymology, VOlume III 1957 London: Academic Press 974 977

[B47] Zhang X Broderick M Amperometric detection of nitric oxide Mod Asp Immunobiol 2000 1 4 160 165

[B48] Sambrook J Fritsch EF Maniatics T Molecular cloning: a laboratory manual 1989 New York: Cold Spring Harbor Laboratory Press

[B49] Glenn SA Gurich N Feeney MA Gonzalez JE The ExpR/Sin quorum-sensing system controls succinoglycan production in *Sinorhizobium meliloti* J Bacteriol 2007 189 19 7077 7088 10.1128/JB.00906-07 17644606 PMC2045190

[B50] Krol E Becker A Global transcriptional analysis of the phosphate starvation response in *Sinorhizobium meliloti* strains 1021 and 2011 Mol Genet Genomics 2004 272 1 1 17 15221452 10.1007/s00438-004-1030-8

